# Vascular Risk Factors in Patients with Different Subtypes of Ischemic Stroke May Affect Their Outcome after Intravenous tPA

**DOI:** 10.1371/journal.pone.0131487

**Published:** 2015-08-06

**Authors:** Yi Dong, Wenjie Cao, Jinma Ren, Deepak S. Nair, Sarah Parker, Jan L. Jahnel, Teresa G. Swanson-Devlin, Judith M. Beck, Maureen Mathews, Clayton J. McNeil, Manas Upadhyaya, Yuan Gao, Qiang Dong, David Z. Wang

**Affiliations:** 1 Department of Neurology, Huashan Hospital, State Key of Laboratory of Neurobiology, Fudan University at Shanghai, Shanghai, China; 2 Center for Outcomes Research, University of Illinois College of Medicine at Peoria, Peoria, IL, United States of America; 3 INI Stroke Network, OSF Healthcare System, Department of Neurology, University of Illinois College of Medicine at Peoria, Peoria, IL, United States of America; 4 Department of Neurology, The First Affiliated Hospital of Zhengzhou University, Zhengzhou, Henan, China; St Michael's Hospital, University of Toronto, CANADA

## Abstract

Intravenous (IV) tissue-type plasminogen activator (tPA) is the only approved noninvasive therapy for acute ischemic stroke (AIS). However, after tPA treatment, the outcome of patients with different subtypes of stroke according to their vascular risk factors remains to be elucidated. We aim to explore the relationship between the outcome and different risk factors in patients with different subtype of acute strokes treated with IV tPA. Records of patients in this cohort were reviewed. Data collected and analysed included the demographics, vascular risk factors, baseline National Institutes of Health Stroke Scale (NIHSS) scores, 90-day modified Rankin Scores (mRS), and subtypes of stroke. By using the 90-day mRS, patients were dichotomized into favorable versus unfavorable outcome in each subtype of stroke. We identified the vascular risk factors that are likely associated with the poor outcome in each subtype. Among 570 AIS patients received IV tPA, 217 were in the large artery atherosclerosis (LAA) group, 146 in the small vessel occlusion(SVO) group, and 140 in the cardioaortic embolism(CE) group. Lower NIHSS score on admission was related to favorable outcome in patients in all subtypes. Patients with history of dyslipidemia were likely on statin treatment before their admission and hence less likely to have elevated cholesterol level on admission. Therefore, there was a possible paradoxical effect on the outcome in patients with LAA and SVO subtypes of strokes. SVO patients with history of diabetes had higher risk of unfavorable outcome. SVO patients had favorable outcome if their time from onset to treatment was short. In conclusion, the outcome of patients treated with IV tPA may be related to different vascular risk factors associated with different subtypes of stroke.

## Introduction

Tissue-type plasminogen activator (tPA) is the only approved intravenous (IV) therapy for acute ischemic stroke (AIS) [1]. The National Institute of Neurological Disorders and Stroke (NINDS) trials have demonstrated the efficacy of recombinant tPA treating AIS of all subtypes within 3 hours of stroke onset.[2–5] One cohort study reported that the rate of complete recanalization at 6 hours was higher (P = 0.006) in patients with cardioaortic embolic (CE) stroke compared to those with other stroke subtypes [6]. The Helsinki Stroke Thrombolysis Registry Group conducted a retrospective study on stroke subtypes and the outcome of these patients after thrombolysis.[7] In that study, the risk factors were equally distributed in all subtypes of strokes and it was found that patients with small-vessel disease strokes were likely to have a good outcome. Other studies have examined only the risk factors that may influence the outcome post IV tPA but did not study further to look into the influence of each subtype of stroke with the risk factors on the outcome. In these studies, diabetes mellitus have been identified as a common risk factor associated with unfavorable outcome.[8] Therefore, it remains unclear if different subtypes of stroke with different severity and associated risk factors would have similar outcome post IV tPA treatment. In this study, we evaluated factors that may affect the outcome post IV tPA in patients with different subtypes of stroke associated with different risk factors, their stroke severity and onset to treatment time (OTT).

After adjusting for patient’s baseline characteristics, we performed further statistical analysis to determine if an association is present between stroke subtypes plus the risk factors that would impact the outcome after tPA treatment. The hypothesis was that the outcome from the treatment could be influenced by stroke subtypes along with co-existing risk factors. In addition, OTT was used to help study the recognition time of stroke symptoms and the time of treatment, which may influence the outcome.

## Materials and Methods

### Design and Patients

This was a retrospective observational study. We identified all patients with AIS who received IV tPA only at OSF Saint Francis Medical Center, a Joint Commission certified Comprehensive Stroke Center (CSC) in Peoria, Illinois between March 2010 and October 2013.

As a certified CSC, we provide the highest level of stroke care 24 hours and 7 days a week. Two types of patients who receive IV tPA were entered into the analysis: those treated in our emergency room (ER) and those who used “drip and ship” method and were transferred to our center after IV tPA has been started or given. The European cooperative acute stroke study (ECASS) III criteria were used to select patients for IV tPA treatment [9]. The dose of IV tPA was the standard dose used per package insert, which was 0.9 mg/kg, maximum dose of 90 mg, giving 10% as an IV bolus over one minute, and the remaining via IV drip over one hour.

All patients were admitted within 24 hours post treatment. Diagnosis of AIS was confirmed by both clinical symptoms and neuroimaging studies such as a non-contrast computer tomography (CT) or magnetic resonance imaging (MRI) of brain with diffusion weighted sequences. Data obtained include demographic information and vascular risk factors such as hypertension, diabetes, hyperlipidemia and smoking history. Dyslipidemia was defined as one or more abnormal levels of triglyceride, cholesterol, low density lipid protein cholesterol and high density lipid protein cholesterol. Patients were given the diagnosis of dyslipidemia if they were on any lipid lowering agent on admission. Diagnosis of hypertension and diabetes mellitus were also made according to the history on admission. All data was obtained from electronic health record and de-identified by M.C. prior to analysis. The Peoria community Institutional Review Board has approved this study.

### Clinical Assessment and Follow-Up

NIHSS scores were recorded on presentation and discharge by two neurologists. This was because the admitting neurologist was different from the one discharging the patient. According to the medical records, the initial NIHSS scores were collected on stroke patients in our ER and ERs where “drip and ship” started. We used the Safe Implementation of Treatments in Stroke Monitoring Study (SITS-MOST) definition to determine symptomatic intracranial hemorrhage (sICH) [10].

The primary outcome was the modified Rankin Scale (mRS) score, which was obtained during the 3-month follow-up visit in the stroke clinic (mean follow-up time 2.7 ±0.6 months). According to ECASS III and Canadian Alteplase for Stroke Effectiveness Study (CASES) trial [9,11], favorable outcome was defined as an mRS score of 0 or 1 at the follow-up visit, while unfavorable outcome was defined as an mRS score between 2–6.

Stroke etiology was classified according to the Stop Stroke Study Trial of Org 10172 in Acute Stroke Treatment (SSS-TOAST) at discharge [3,12]. All patients enrolled in this study were classified into these subtypes: large artery atherosclerosis (LAA), small-vessel occlusion (SVO), cardioaortic embolism (CE), and stroke of other determined etiology and stroke of undetermined etiology (OE). All supplement data was online (seen S1 Dataset).

### Statistical Analysis

Statistical analysis was performed by using Version 20 SPSS (SPSS Inc, Chicago, USA). Statistical significance was defined as a two-tailed with a P≤0.05 level. Patients were categorized into three subtypes of stroke by using the follow-up mRS scores: LAA, SVO and CE. Since the mechanism of stroke was unclear in OE subtype (small sample size), it was difficult to associate it with the risk factors. Therefore, the OE group was not included in any further analysis.

Variables were reported as mean±standard deviation, median and range for continuous and ordinal variables, and percentage for categorical variables. The continuous variables were analyzed by ANOVA. Ordinal variables were analyzed by Student t-test. In univariate analysis, the associations between different subtypes of stroke and outcomes were examined by using multivariable logistical regression analysis and adjusted for age, sex, race, OTT, NIHSS scores, and history of smoke, hypertension, diabetes, atrial fibrillation and stroke. Logistic regression was used to analyze risk factors of outcomes in different subtype of stroke patients.

## Results

Between March 2010 and October 2013, 570 AIS patients were treated with IV tPA. Among them, 40 had additional intra-arterial tPA; 20 had thromboembolectomy; 2 had middle cerebral artery stents. In addition, during the acute treatment phase, 11 had carotid artery endarterectomy (CEA) and 6 had carotid artery stenting (CAS). During the hospitalization, 5 had myocardial infarction (MI) and required percutaneous coronary intervention (PCI); one had a stent for deep venous thrombosis (DVT). Only 485 patients (85.1%) were qualified to be included in this study. Among them, 264 (46.3%) patients achieved favorable outcome during their three months follow-up (Table 1). Seventeen (3.5%) patients had asymptomatic ICHs and 4(0.8%) had sICHs. Two patients had other major bleeding events.

**Table 1:** Characteristics of acute stroke patients with intravenous tPA at baseline.

Items	n	% or mean
**Age, years old, mean(SD)**	570	70.6(13.0)
**Female**	284	49.8%
**Race**		
White	511	89.6%
Black	20	3.5%
UTD	39	6.8%
**History**		
AF	83	14.6%
HTN	461	80.3%
Stroke	115	20.4%
Smoking	286	50.2%
Diabetes	191	33.5%
Dyslipidemia	216	37.2%
Pre-statin*	237	41.6%
Admission triglyceride(SD)	447	135.2(95.3)
Admission-LDL>100dl/ml	211	37.0%
Both diabetes and dyslipidemia	97	17%
**Admission NIHSS, median(IQR)**	560	7(4–15)
**Discharge NIHSS median(IQR)**	573	1(0–5)
**Toast subtype**		
LAA	217	38.1%
SVO	146	25.6%
CE	140	24.6%
OE	67	11.8%
**Anticoagulation**		
Aspirin	220	38.6%
Clopidogrel	150	26.3%
Aggrenox	14	2.5%
Warfarin	51	8.9%
LMWH or other	56	9.8%
**OTT(min), median(IQR)**	483	120(81–168)
**mRS, median(IQR)**	570	2(0–4)
0	168	29.5%
1	96	16.8%
2	57	10.0%
3	71	12.5%
4	67	11.8%
5	43	7.5%
6	68	11.9%

tPA = tissue-type plasminogen activator; SD = standard deviation; UTD = undetermined; AF = atrial fibrillation; HTN = hypertension; NIHSS = National institute of health stroke scale; LAA = large artery atherosclerosis; SVO = small vessel occlusion; CE = cardioaortic embolism; OE = stroke of other determined etiology and stroke of undetermined etiology; LMWH = low molecule weighted heparin; OTT = onset to treatment time; min = minutes; IQR = interquartile; mRS = modified Rankin Scale.

*24 patients without a history of dyslipidemia were treated with statin before admission.

Two patients with a history of dyslipidemia were not on statin. Fifty six patients onstatin still had a high level of LDL on admission.

Among 485 patients only received IV tPA, 217 (35.5%) were classified as LAA group, 144 (29.7%) as SVO group, 104 (21.4%) as CE group, and 21(13.8%) as unknown or with other etiology. The relationship between subtypes of stroke and outcome were analyzed after adjustment for age, gender, OTT, NIHSS scores on admission, history of stroke, hypertension, diabetes mellitus (DM), dyslipidemia, atrial fibrillation and stroke (Table 2). According to their mRS scores, an obvious difference in outcomes among different subtypes was observed. Even after adjusted for baseline and risk factors, the trend still exists (p = 0.06).

**Table 2:** Comparison of outcomes among different TOAST subtypes.

	mRS 0–1,N (%)	Unadjusted analysis			*Adjusted analysis		
		OR	95%CI	P value	OR	95%CI	P value
LAA(n = 1 2)	72(41.8)	0.85	0.47–1.53	0.58	1.29	0.61–2.71	0.51
SVO(n = 1 4)	89(61.8)	1.91	1.04–3.50	0.04	2.11	0.98–4.52	0.06
CE(n = 108)	47(43.5)	0.91	0.48–1.71	0.76	1.38	0.51–3.73	0.73
OE(n = 61)	28(45.9)	ref	-		ref	-	-

*Adjusted OR was calculated by using multivariable risk-adjustment model adjusted for age, sex, onset to treatment time,admission NIHSS scores, history of smoking, history of hypertension, diabetes, atrial fibrillation, dyslipidemia and stroke.

TOAST = Trial of Org 10172 in Acute Stroke Treatment; mRS = modified Rankin Scale; OR = odd ratio; CI = confidential interval; LAA = large artery atherosclerosis; SVO = small vessel occlusion; CE = cardioaortic embolism; OE = stroke of other determined etiology and stroke of undetermined etiology; Ref = reference.

The multivariable logistic regression analysis was summarized (Table 3). Lower NIHSS scores on admission were related to favorable outcome in patients with all subtypes (LAA group p<0.001,SVO group p = 0.003, CE group p = 0.006 respectively). SVO patients with history of diabetes had higher risk of unfavorable outcome [SVO group OR 0.31, 95%CI (0.15–0.85), p = 0.02]. SVO patients would have favorable outcome when their OTT [OR 0.99, 95%CI 0.99–1.000, p = 0.05] time was shorter.

**Table 3:** Risk factors of outcomes in different subtype of stroke patients in multivariable logistic regression.

		LAA(n = 172)						SVO(n = 144)						CE(n = 108)				
	mRS 0-1(n = 72)	mRS>1(n = 100)	Sig	Exp(B)	95% C.I.		mRS 0–1(n = 89)	mRS>1(n = 57) (n = 55)	Sig	Exp(B)	95% C.I.		mRS 0–1(n = 47)	mRS>1(n = 61)	Sig	Exp(B)	95% C.I.	
**Age(mean, Y.O)**	69.8±13.0	71.2±12.6	0.62	1.01	0.97	1.05	67.1±12.4	69.5±14.4	0.50	1.001	0.98	1.05	74.4±11.1	74.4±13.9	0.86	1.00	0.96	1.04
**Gender(mle)**	33(45.8%)	48(48%)	0.53	0.75	0.31	1.84	54(60.7%)	27(49.1%)	0.51	1.35	0.56	3.26	29(61.7%)	32(52.5%)	0.96	1.02	0.37	2.83
**OTT(mea+SD,Min)**	134.2+62,9	136.6+71.9	0.90	1.00	0.99	1.01	127.1+60.3	158.3+74.1	**0.05**	1.01	1.00	1.01	114.6+66.4	130.0+63.7	0.35	1.00	1.00	1.01
**AdmiNIHmean+SD,point)**	5.6+4.2	13.5+8.1	<0.001*	1.29	1.17	1.43	4.3+4.3	7.1+6.3	0.003*	1.16	1.05	1.29	8.1+7.0	12.9+6.7	0.006*	1.11	1.03	1.20
**hxlipid(w/)**	43(59.7%)	37(37%)	0.05	2.61	1.00	6.80	61(68.5%)	37(67.3%)	0.05	0.35	0.12	1.02	32(68.1%)	37(60.7%)	0.381	1.59	0.57	4.44
**HxHTN(/)**	19(26.4%)	15(15%)	0.33	1.77	0.56	5.57	27(30.3%)	8(14.5%)	0.39	171	0.50	5.83	5(10.6%)	9(14.8%)	0.33	0.47	0.10	2.16
**HxDM(w/)**	51(70.8%)	58(58%)	0.39	1.50	0.59	3.82	68(76.4%)	31(56.4%)	0.02*	3.21	.1.18	8.71	35(74.5%)	36(59.0%)	0.12	2.41	0.81	7.16
**Hxstroke(/o)**	59(81.9%)	76(76%)	0.42	0.64	0.22	187	72(80.9%)	39 (70.9%)	0.70	1.24	0.43	3.61	38(80.9%)	48(78.7%)	0.54	1.47	0.43	4.97
**Hxsmoke(/o)**	38(52.7%)	45(45%)	0.67	1.22	0.50	2.98	48(57.2%)	32(58.2%)	0.38	0.66	0.26	1.68	20(42.5%)	35(57.4%))	0.25	0.56	0.21	1.51

LAA group, 19 patients missing history of dyslipidemia, 18 patients missing history of atrial fillibration; SVO group, 3 patients missing history of dyslipidemia,3 patients missing history of atrial fillibration; CE group, 14 pts missing history of dyslipidemia,12 patients missing history of atrial fillibration. LAA = large artery atherosclerosis; SVO = small vessel occlusion; CE = cardioaortic embolism; mRS = modified Rankin Scale; CI = confidential interval; LAA = large artery atherosclerosis; SD = standard deviation; Admis NIH = NIHSS scores at admission.

Patients with history of dyslipidemia produced a paradoxical impact on their outcome, since they had lower levels of low-density lipoprotein (LDL), while patients without previous statin treatment had higher levels of LDL[OR 2.12, 95%CI 1.61–2.79, p<0.05]on admission. We therefore analyzed the relationship between history of dyslipidemia and LDL levels(more than 100dl/ml) and found that the statin treatment might play a key role in patients with history of dyslipidemia. On admission, 24 patients without a history of dyslipidemia were on statins, 2 patients with a history of dyslipidemia were not on statin and 56 patients still had high levels of LDL despite being on treatment. We found that patients with history of dyslipidemia were more likely to be on statin treatment (OR 0.06, 95% CI 0.04–0.09, p<0.01). Patients in LAA subtype had a higher incidence of dyslipidemia (p = 0.057) and was associated with a higher risk of unfavorable outcome[OR 0.68, 95%CI 0.14–1.00, p = 0.05]. A trend of lower risk of unfavorable outcome was also found in SVO group[OR 0.35 95% 0.98–8.33,p = 0.05] and CE group. Therefore, it seems that treating LDL levels was not as beneficial for patients in LAA group.

## Discussion

IV-tPA is effective in treating patients with all types of AIS within 4.5 hours [1]. Some studies reported no obvious differences in outcome regardless of stroke subtypes [2–5] but the impact of risk factors on the outcome of these patients of different subtypes has not been well explored. Our study has found that different risk factors have an effect on outcome of AIS patients of different subtypes post IV tPA.

OTT time is critical to the success of iV tPA treatment. Several large trials, such as NINDS [1], ECASS-III [9], and SITS-MOST [10] have showed that the earlier IV tPA was given, the better the outcome. However, our study only showed such benefit of shortened OTT in those patients with SVO subtype of stroke. There are several possible explanations for this phenomenon. Patients with LAA or CE subtypes usually were brought to the ER quicker because of easier recognition of symptoms of a more severe stroke. Therefore, the benefit of short OTT time would not be as evident. However, SVO patients probably presented to the ER later rather than sooner because of milder symptoms. These patients might think that their symptoms would subside spontaneously so they would wait rather than go to ER. The treating physicians would often hesitate to consider IV tPA treatment in these patients because of milder symptoms. Hence, the benefit of faster treatment times (129±60 minutes in patients with favorable outcome vs. 157±73 minutes in patients with unfavorable outcome) would only become more apparent in SVO patients [13].

In general, the severity of neurological deficit, measured by NIHSS score on admission, is directly related to patients’ outcomes. In our study, NIHSS score on admission had an impact on the outcome of patients of all subtypes. The issue of which subtype of stroke responds better to IV tPA has been conflicting in the literature. One report stated that moderate to severe strokes benefit more from IV-tPA than those with mild strokes. The same study also confirmed that CE infarctions might benefit more from IV tPA than other etiology [14]. Another study concluded that complete recovery was likely in patients who were younger and male, had milder stroke symptoms, and did not have CE type of strokes [15]. Our finding supports that NIHSS score on admission could be predictive of outcome and shorter OTT time is the key to successful thrombolysis.

One study showed that dyslipidemia affected the outcome of patients after receiving IV tPA [16]. Its adverse effect could be related to the formation of non-dissolvable lipid rich thrombus, which could in turn cause larger infarction and hemorrhagic transformation. However, our study demonstrated a paradoxical effect of dyslipidemia on the outcome post IV tPA. This phenomenon was difficult to explain but was supported by the findings in a sub-study of CHANCE trial. CHANCE has found that patients with minor strokes and well-controlled glucose more likely had a history of dyslipidemia but similar LDL levels compared to those with poor controlled glucose at admission. They also had low rate of recurrent strokes[17]. In our study, LAA patients with dyslipidemia had less favorable outcome than those in SVO and CE group, indicating the complexity of LAA stroke and the cause of it could be multifactorial. Furthermore, treatment of dyslipidemia with statins showed a trend of benefit for patients with SVO. In LAA and CE group, the protective effects of statins could have been nullified by increased incidence of hemorrhagic transformation in our study.

Diabetes is a common risk factor of stroke. Hyperglycemia at the onset of acute stroke may adversely affect the outcome. In SITS-MOST, IV tPA-treated patients with DM and previous stroke showed less chance of improvement, lower rate of functional independency, and a higher rate of mortality [9]. Our study certainly confirmed these findings. However, in our study the adverse impact on recovery by DM only was seen in patients with SVO subtype, a rather surprise finding. DM causes chronic micro vascular damage and predisposes vessels to the risk of rupture. DM is also associated with diminished fibrinolysis capacity and increased concentration of serum plasminogen activator inhibitor-1 [18]. Patients with DM often display impaired response to antithrombotic treatment [11]. It is still debatable how glucose affects stroke. Two published studies demonstrated that hyperglycemia might have improved odds of a favorable outcome in lacunar stroke, but worsening odds in non-lacunar stroke[19–20]. In addition, type II diabetes is related to worsening of functional outcome.[21] However, recent report of glycated albumin level could be a potential biomarker of the effect of antiplatelet therapy in patients with minor stroke or TIA[17,22]. Patients with lower level of glycated albumin had more recurrent strokes[17].

Our study only had 4 (0.8%) cases of sICH. Comparing to the findings of 4% of sICH in the International Stroke Trial (IST-3),[23] our rate was sICH was very low. This was likely related to the strategies of improving processes to offer treatment early and following the treatment protocols. Some of our patients received IV tPA in the CT scan room.

Our study has several limitations. First, this study was a report of a single center experience. Our retrospective results need to be confirmed in prospectively designed studies. Second, no NIHSS score was documented on admission in 20 patients. Third, analysis could be biased since some LAA subtype patients were excluded because they had additional interventional treatment. Forth, subtypes of stroke in patients who have had stroke in the past were not available. This information could have an impact on the outcome. Finally, in our cohort 12.7% of all strokes were classified as other or undetermined subtypes and were excluded. Patients in this category had the highest rate of vascular risks.

## Conclusion

Vascular risk factors may determine the outcome in patients with different subtypes of acute ischemic stroke after IV-tPA treatment. Dyslipidemia created a paradoxical effect, in that patients with LAA would have a worsening of outcome compared to those with SVO. DM has an unfavorable impact on the outcome for SVO and CE patients. Our finding may assist clinicians calculating prognosis for patients who need IV tPA therapy.

## Supporting Information

S1 Dataset Database of patients treated with tissue plasminogen activator. (SAV)
